# The Role of Gaze in Shaping Forms of Reflective Practice: A Legitimation Code Theory Analysis of Clinical Supervision in Danish Primary Care

**DOI:** 10.1111/scs.70176

**Published:** 2026-01-06

**Authors:** Anette Lykke Hindhede, Helle Hindhede Hald, Christina Andersen

**Affiliations:** ^1^ Department of Public Health University of Copenhagen Copenhagen Denmark; ^2^ UCSF: Center for Health Research, Copenhagen University Hospital Copenhagen Denmark; ^3^ Forensic Psychiatric Clinic, Ministry of Justice Copenhagen Denmark

**Keywords:** clinical supervision, gaze, Legitimation Code Theory, primary care, reflective practice

## Abstract

**Aims and Objectives:**

This study aimed to investigate how supervision models may cultivate or constrain different ways of knowing and learning in primary care.

**Methodological Design and Justification:**

The research employed a qualitative methodological design, grounded in Legitimation Code Theory, to gain an in‐depth understanding of the dynamics at play within various supervision models. It aligns with the QRSR guidelines.

**Ethical Issues and Approval:**

Ethical considerations were thoroughly addressed, and approval was obtained prior to initiating the study, ensuring participant confidentiality and informed consent.

**Research Methods, Instruments, and Interventions:**

The study utilised qualitative interviews as the primary research method, conducting 18 interviews with a diverse range of healthcare professionals, including leaders, nurses, nursing assistants, physiotherapists, and both internal and external supervision consultants.

**Outcome Measures:**

The analysis focused on identifying how different supervision models influenced reflective practice and shaped the participants' perceptions regarding the effectiveness and utility of these models.

**Results:**

Findings illustrated the complex interplay of cultivated, social, and trained gazes within healthcare settings, highlighting how different forms of legitimation shape what counts as meaningful understanding and reflective practice.

**Study Limitations:**

While the study provides valuable insights, it is important to acknowledge limitations related to the heterogeneous nature of the material across the interventions.

**Conclusions:**

The concept of gaze not only elucidates the presuppositions underlying different supervision models but also elucidates how the usefulness of different supervision models is legitimated within practice.

## 1 Introduction

Healthcare systems are undergoing significant transformations that prioritize reducing hospital stays and shifting care to primary care settings [1]. Consequently, there is an increased emphasis on developing innovative primary care delivery models [2, 3, 4, 5] and fostering diverse interprofessional teams to ensure high‐quality health care services [6, 7, 8, 9]. These trends are evident across European healthcare systems as they adapt to evolving patient needs and aim for integrated care solutions [10, 11].

Reflection on one's own practice is central when the healthcare system requires development. Professionals operating within the complex context that the healthcare system represents face challenges that demand an ability for continuous adaptation [12]. Reflection on one's own practice is a method through which professionals have the opportunity to analyse, evaluate, and interpret, thereby identifying strengths, weaknesses, and potential areas for improvement [13, 14, 15]. Reflection and reflective practice originate from the works of influential thinkers in the fields of education and professional practice, such as John Dewey and Donald Schön. Dewey defined reflection as “active, persistent, and careful consideration” of beliefs supported by evidence [16], while Schön expanded on this with the concept of “reflection‐in‐action” [13]. Reflective practice involves a systematic enquiry aimed at enhancing our understanding of professional practice [17]. A key component is the ability to navigate emotionally challenging situations in practice [18, 19, 20, 21]. Critically reflective practice emphasises awareness of emotional responses, challenges assumptions, and examines personal values and power dynamics in relationships, fostering reflexivity about one's context and its impact on daily practice [17, 22].

To promote reflexivity, educational initiatives such as supervision are increasingly incorporated into health professional training programs [23, 24, 25, 26, 27, 28, 29]. For instance, nursing students are often tasked with reflective assignments, including essays, reports, journals, logs, and portfolios [30, 31, 32] as ways of examining and considering alternative viewpoints that may contrast with established norms [33].

Learning through reflection is shown to correlate positively with self‐reported competence levels among both novice and experienced nurses. Takase et al. [34] found that less experienced nurses benefited from learning through practice and collaboration with colleagues, while experienced nurses benefited additionally from constructive feedback and targeted training related to their self‐reported competencies [34].

However, despite the recognised importance of reflective practices, several barriers hinder their implementation in clinical settings. Factors such as time constraints, misaligned educational expectations, and limited access to supervision complicate practitioners' engagement in these reflective processes [35, 36]. Moreover, the effects of interventions aimed at promoting critical thinking and clinical decision‐making skills among nursing students remain inconclusive [37, 38, 39, 40].

### 1.1 Supervision to Improve Reflective Practices

Reflective practice has been widely discussed in health professions education as a means to address the complexity of clinical situations [41, 42, 43]. It is commonly conceptualised as an active process in which individuals examine their thoughts, beliefs, and actions in relation to new experiences and evidence [44]. Studies suggest that such processes can support professional development and informed decision‐making [18]. Supervision has been described in the literature as a formalised setting for guided reflection, typically facilitated by a more experienced practitioner or supervisor [45]. Within this context, reflective questions and frameworks are often employed to promote the supervisee through structured reflection. When supervision takes place in clinical settings, it is frequently referred to as clinical supervision [46], a term adopted in this paper.

Empirical studies indicate that clinical supervision differs from informal, ad hoc consultations by being anchored in specific professional challenges and organised within a pedagogical framework [47]. This structured approach is intended to ensure continuity and depth in learning processes, distinguishing it from everyday exchanges [48].

To enhance reflective learning, it is important to understand how knowledge is constructed, valued, and legitimated within supervision practices [49]. In the municipal project we evaluated, four distinct models of supervision emerged: Structured team supervision, reflective clinical practice supervision, skills development supervision, and communication skills enhancement supervision. These are not established models but rather variations observed in practice, each reflecting different pedagogical orientations and assumptions about what constitutes valuable learning.

Legitimation Code Theory (LCT) offers a productive lens for analysing these emergent models because it focuses on the organising principles that shape knowledge practices [49]. Instead of treating supervision as a uniform activity, LCT enables us to examine the epistemic and social dimensions embedded in supervision, which often remain implicit. By applying LCT, this study aims to make visible the codes that underpin these supervision practices, offering insights into potential challenges in their implementation and opportunities for more coherent pedagogical strategies. To lay the groundwork for this analysis, we now introduce LCT as a framework for understanding the complexity of teaching and learning within these emergent supervision models.

### 1.2 Legitimation of Knowledge and Knower Practices

This qualitative study is grounded in a critical realist paradigm, which posits that an ontologically real world exists independently of our perceptions, while epistemic access to that reality is mediated through socially and historically situated knowledge practices. Knowledge is therefore considered fallible and contingent, yet capable of generating plausible explanations of underlying causal mechanisms and structures [50, 51, 52]. This epistemological stance informs our analysis of how supervision practices function as sites where particular forms of knowing and being are legitimised and reproduced within social contexts.

This concern with the structuring of knowledge aligns with LCT, developed by Karl Maton in the early 2000s, and builds on Bernstein's sociology of education [53] and Bourdieu's field theory [50, 54]. LCT offers a set of conceptual tools for analysing the organising principles of knowledge practices across disciplines. It has been widely applied in studies of curriculum design [55], professional education [56], patient education [57], and disciplinary knowledge structures [58, 59], including fields such as nursing [60], and engineering [61]. While LCT has not yet been used in research on clinical supervision, its concepts have informed studies in health professions education by examining epistemic and social relations in learning environments [62, 63]. This makes LCT particularly relevant for our study, as it offers a systematic framework for analysing how supervision practices shape access to legitimate knowledge and legitimate ways of being a knower within contexts such as continuing professional development (CPD) [64].

### 1.3 Core Dimensions and the Concept of Gaze

LCT conceptualises knowledge practices through two organising principles:

- Epistemic relations (ER): The degree to which legitimacy depends on possessing specialised knowledge, skills, or procedures.
- Social relations (SR): The degree to which legitimacy depends on attributes of knowers (their dispositions, identity, or ways of being).

To analyse SR in more detail, LCT distinguishes two relational aspects:

- Subjective relations (SubR): How far legitimacy depends on personal attributes or identity markers (e.g., innate qualities, social category).
- Interactional relations (IR): How far legitimacy depends on ways of interacting with others and engaging with recognised practices.

These aspects form what Maton calls the social plane, which maps how knower legitimation varies according to SubR and IR. This plane allows us to identify different orientations toward what counts as a legitimate knower.

Within this framework, gaze refers to a socially recognised way of seeing and acting that signals legitimate knower status [65]. Gazes are not neutral; they structure access to valued practices and shape how fields reproduce or transform. Maton identifies four principal gazes:

- Born gaze: Legitimacy is associated with innate qualities or “natural talent.” This gaze positions SubR as comparatively stronger and IR as relatively stronger as well, emphasising an inherent or naturalised way of being a knower.
- Social gaze: Legitimacy derives from belonging to particular social categories (e.g., gender, class, ethnicity). Here, SubR are relatively strong, while IR are comparatively weaker, foregrounding identity‐based criteria over learned ways of knowing.
- Cultivated gaze: Legitimacy depends on dispositions developed through sustained immersion and structured engagement with valued practices. In this case, SubR are comparatively weaker, whereas IR are relatively stronger, highlighting how knower legitimacy is acquired through prolonged exposure and participation.
- Trained gaze: Legitimacy rests on the mastery of codified procedures through formal training. Both SubR and IR are positioned as relatively weaker compared to other gazes, with emphasis placed on technical competence rather than interpretive or identity‐based criteria.

Mapping gazes on the social plane enables us to trace movements from identity‐based legitimation (born or social gazes) toward forms grounded in dispositions and practices developed through structured interaction (cultivated or trained gazes). This analytical lens is crucial for understanding supervision, as it reveals how different supervisory models privilege particular knower orientations and thereby shape possible pathways for professional development.

Supervision practices do not operate in a vacuum; they reproduce institutional codes that define what counts as legitimate knowing and being. In LCT terms, these orientations are expressed through gazes; that is socially recognised ways of perceiving, valuing, and acting that confer knower legitimacy. Supervisors act as gatekeepers of these norms, while participants navigate and negotiate them in their effort to gain recognition.

### 1.4 Analytical Aim

**FIGURE 1:**
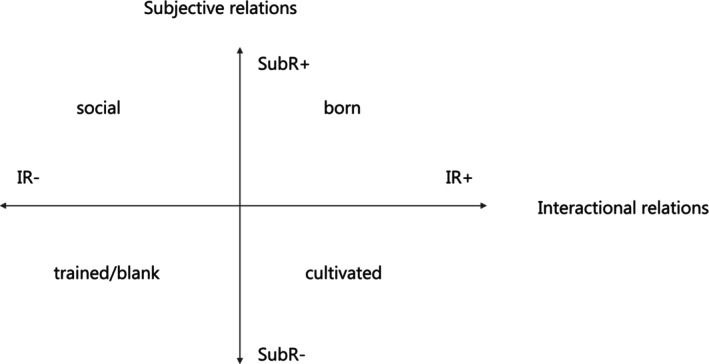
The social plane‐gazes Maton (41: 186).

Our analysis investigates how different supervision models privilege particular knower orientations by identifying the dominant gaze within each model and tracing possible movements across the social plane (see Figure 1). Drawing on SubR and IR as analytic lenses, we examine how these relational configurations open up or constrain opportunities for reflective practice and professional development [65].

## 2 Methods

This qualitative study adheres to the Standards for Reporting Qualitative Research (SRQR) to ensure transparency and rigor in the research process [66]. The study evaluated a municipal project aimed at strengthening professional quality and fostering a more reflective and supervisory approach to practice within primary care in a large Danish city. External supervisors were appointed by the municipality to facilitate the interventions.

The project followed different supervision approaches to explore their applicability across contexts and to identify the pedagogical frameworks and competencies required for each. Interventions were demand‐driven, meaning that participating units self‐selected their supervision focus based on locally identified needs. Through this iterative process, four distinct supervision models emerged:

- Structured team supervision.
- Reflective clinical practice supervision.
- Skills development supervision
- Communication skills enhancement supervision

These supervision models were not predefined models but practice‐based variations that developed during implementation. Supervision sessions varied in length, frequency, and organisational context (see Table 1), reflecting the diversity of local needs and conditions.

**TABLE 1:** Description of the four supervision interventions.

Intervention	Description
Structured team supervision (Intervention 1)	*Purpose*: A model with a clear citizen‐oriented and organisational goal *Duration*: Sessions of 1 h, initially incorporating courses and workshops for all employees, transitioning to short sessions (‘15 professional minutes’) with key individuals *Participants*: Interdisciplinary model with social and health assistants, nurses, and physiotherapists (closed groups) *Content*: Focused on concrete cases, professional themes, and self‐chosen topics related to mental vulnerability *Who runs it*: External consultant, with key individuals responsible for continuity post‐intervention
Reflective clinical practice supervision (Intervention 2)	*Purpose*: A model with a clear organisational goal focused on improved practice, integration of new colleagues, and collaboration *Duration*: Sessions (3 × 1 h) *Participants*: Monodisciplinary model in two smaller groups of approximately 6 nurses (closed groups) *Content*: Focused on specific tasks, professional themes, and self‐chosen topics *Who runs it*: Two external consultants for each of the two nurse groups
Skills development supervision (Intervention 3)	*Purpose*: A model with a clear organisational goal concentrating on the role of group leaders as facilitators *Duration*: Sessions with workshops *Participants*: Monodisciplinary model with group leaders from home care and home nursing (closed groups) *Content*: Focused on professional themes and ‘AGB’ as a tool *Who runs it*: External consultant hired for training and support
Communication skills enhancement supervision (Intervention 4)	*Purpose*: To provide experts with practical tools and methods through communicative simulation to enhance the training of professional skills among colleagues *Duration*: Sessions (3 × 3 h across 3 days), conducted digitally *Participants*: Experts in dementia, including nurses, occupational or physiotherapists, and educators (closed groups) *Content*: Communicative simulation training focused on enhancing guidance skills *Who runs it*: External consultant for simulation training

The study utilised purposefully selected participants representing various roles within the healthcare context to provide in‐depth insights into each intervention. In total, 18 semi‐structured interviews were conducted, including both individual and focus group formats. Criteria for inclusion were based on participants' involvement in the interventions and relevant professional experience. Sampling saturation was reached by confirming that no new themes emerged from the interviews.

The first author conducted all interviews for Interventions 1, 2, and 3, while a research assistant conducted the interviews for Intervention 4. The authors, experienced in health professions education and qualitative research, maintained reflexivity by discussing preconceptions and potential biases in regular meetings.

*Structured team supervision* (*Intervention 1*): Six interviews were conducted with eight staff members, comprising five individual interviews and one focus group interview. Participants included a physiotherapist, two assistant nurses, a nurse leader, and two key personnel (one assistant nurse and one nurse). All interviews were conducted by the first author. Additionally, an observation of a supervision session was conducted.

*Reflective clinical practice supervision* (*Intervention 2*): A focus group interview was conducted with two home care nurses, alongside an individual interview with one of the two external consultants (hired as a development consultant in a private consulting firm) who led the supervision sessions.

*Skills development supervision* (*Intervention 3*): Two qualitative interviews were conducted with two group leaders, one with a nursing background and one with an assistant nursing background, who served as “pilots” for the initiative, focusing on their goals, challenges, and experiences. Additionally, an interview with an assistant nurse who participated in the intervention and another with a regional manager (also with a nursing background) were conducted.

*Communication skills enhancement supervision* (*Intervention 4*): This intervention involved participant observation during one online session, followed by an interview with the supervisor (a development consultant from the same private consulting bureau as in Intervention 2). A focus group interview was conducted with two participants (nurses) after their involvement, and a separate interview was held with one leader (nurse) who implemented simulation training in her organization. The interviews in this intervention were conducted by a research assistant.

Each of the 18 interviews lasted between 45 and 90 min, were audio recorded, and transcribed verbatim. The interview guide included themes related to the participants' experiences, perceived challenges, and goals within the context of the interventions. This adaptive approach to data collection allowed for nuanced insights tailored to the specific objectives and contexts of each intervention.

The study was conducted in accordance with applicable GDPR regulations and established research ethics principles, including the Declaration of Helsinki [67]. Informed consent was obtained from all participants prior to the interviews, and participants were assured that their anonymity would be protected. They were also informed of their right to withdraw from the study at any time.

### 2.1 Analytical Strategy

The transcribed interviews and observational notes were imported into NVivo and coded inductively to identify recurring themes related to supervision practices (see Table 2). These initial codes provided a descriptive foundation for understanding variations across the four supervision models.

**TABLE 2:** Analytical framework for measuring the strength of subjective relations (SubR) relative to interactional relations (IR).

Gaze	Subjective relation (SubR)	Interactional relation (IR)	Description
Born gaze	Strong (SubR+)	Strong (IR+)	Legitimacy based on innate qualities or “natural talent”
Social gaze	Strong (SubR+)	Weak (IR−)	Legitimacy based on belonging to a social category (e.g., gender, class, ethnicity)
Cultivated gaze	Weak (SubR−)	Strong (IR+)	Legitimacy depends on dispositions developed through prolonged immersion and structured engagement with valued practices
Trained gaze	Weak (SubR−)	Weak (IR−)	Legitimacy based on mastering codified procedures through formal training; emphasises technical mastery rather than interpretive judgement

Building on this, the analysis was informed by LCT (specifically the concept of gaze) to interpret how supervision practices legitimate particular ways of knowing and being. We examined respondents' accounts of experiences, challenges, and insights with a focus on statements that revealed:

- How knowledge was constructed and displayed.
- How social dynamics influenced learning and supervisory outcomes.

To identify the dominant gaze within each supervision kind, we analysed SR and IR as defined by Maton (50: 184–187). This allowed us to explore whether practitioners were expected to perform, grow, or be supported, and how these expectations shaped their knower orientation.

The strength of each gaze was assessed by evaluating the framing and classification of epistemic and social relations present in the data (see Table 1). In the findings, we present the primary gaze associated with each intervention and discuss potential movements across the social plane in relation to learning, socialisation, and educational transformation. Illustrative quotes from interviews are included to substantiate interpretations.

In the findings, we present the primary gaze of each intervention along with the potential movements across the social plane in the light of the learning, socialisation, and educational transformation. We include quotes from the interviews to support our analysis.

## 3 Findings

The analysis showed that each intervention was associated with a dominant gaze, reflecting different legitimation practices and expectations of practitioners. Across the four supervision models, we identified strong patterns of trained, cultivated, and social gazes, often in combination. Movements across the social plane were observed, reflecting shifts from procedural mastery toward interpretive dispositions, involving strengthened IR and reduced reliance on SubR.

Importantly, we did not find examples of the born gaze, legitimation based on innate qualities or natural dispositions indicating that all interventions relied on learnable and socially mediated forms of professional development rather than assumptions of inherent talent.

Table 3 provides a comparison of the primary and supporting gazes, as well as the supervision types for each intervention and their distinct methodologies aimed at enhancing professional development.

**TABLE 3:** Overview of the gazes of the four supervision models.

Name/intervention	Primary gaze	Supporting gazes
Structured team supervision (Intervention 1)	Social gaze (SubR+, IR−)	Cultivated, trained
Reflective clinical practice supervision (Intervention 2)	Cultivated gaze (SubR−, IR+)	Social, trained
Skills development supervision (Intervention 3)	Trained gaze (SubR−, IR+)	Cultivated, social
Communication skills enhancement supervision (Intervention 4)	Trained gaze (SubR−, IR−)	Cultivated, social

### 3.1 Intervention 1: Structured Team Supervision

This model initially reflected a social gaze, where legitimacy was grounded in shared identity and subjective attributes such as emotional openness (SubR+) with limited structured engagement (IR−). A social and health assistant highlighted the collective challenges they faced: “I think we are much more structured in relation to these teams, where we can meet in team discussions.”

This illustrates how communal engagement conferred legitimacy without strong formal interaction. Emotional openness was emphasised by a leader: “The important part is to listen carefully and recognize when someone is struggling emotionally.”

Such statements show reliance on identity and shared experience rather than structured engagement.

Movement toward a cultivated gaze occurred when dialogic tools and structured practices were introduced, enabling dispositions developed through interaction rather than identity. External consultants also introduced elements of a trained gaze through procedural frameworks, supporting skill development. However, staff expressed reluctance to fully adopt peer‐led models: “It carries a whole different weight if someone from outside comes in with 20 years of experience.” This signals that transitions require institutional endorsement and trust to avoid perceived loss of epistemic authority.

### 3.2 Intervention 2: Reflective Clinical Practice Supervision

Dominated by a cultivated gaze, this model emphasised structured reflection and collaborative dialogue. An external consultant explained: “I create a space where everyone gets to share their experiences.” A home care nurse echoed: “Being able to hear how others manage similar problems fosters a sense of community.”

These remarks show that legitimacy was conferred through dispositions formed via engagement with professional norms rather than innate qualities.

Elements of a trained gaze appeared through procedural scaffolds, creating a hybrid orientation. One participant noted: “I think it's an investment in employees, signaling that we know the psychological work environment is important.”

Movement from trained toward cultivated gaze was supported by dialogic supervision, blending technical frameworks with interpretive judgment.

### 3.3 Intervention 3: Skills Development Supervision

Here, the trained gaze was primary, privileging codified procedures and technical mastery. A group leader stated: “Having a structured way … helped keep our focus.”

This reflects legitimacy tied to operational tools and procedural compliance.

Case‐based discussions introduced partial opportunities for movement toward a cultivated gaze, which requires sustained dialogic engagement and immersion in professional norms rather than occasional case exchanges. As an assistant nurse shared: “It was great to be able to talk about the citizens.”

Such reflections signal the value of applying abstract knowledge to real‐world practice. However, these shifts depended on sustained spaces for dialogue beyond technical training. Without such conditions, legitimation remained tied to operational competence rather than interpretive depth.

### 3.4 Intervention 4: Communication Skills Enhancement Supervision

This intervention also reflected a trained gaze, emphasising task‐based learning and observable behavioural change. A participant remarked:

This training is about cultivating an awareness of how we communicate.

While simulation exercises supported technical proficiency, glimpses of movement toward a cultivated gaze emerged when supervisors encouraged sensitivity to patient context: “They need to listen and catch any signs that help is needed.”

Such movement requires deliberate integration of reflective debriefing and interpretive discussion; otherwise, technical training risks constraining epistemic development.

## 4 Discussion

Across the four interventions, LCT's concept of gaze reveals how different types of supervision foster, limit, or enable movement between ways of knowing and being. Crucially, such transitions across the social plane of gazes are not automatic: they depend on how knowledge and knowers are legitimated, the structuring of supervision spaces, and the institutional values that underpin them.

### 4.1 The Four Supervision Models

Intervention 1, focused on team debriefing for complex cases, initially reflected a social gaze, where legitimacy was grounded in shared identity and emotional openness but weakly structured by formal interaction. This reliance on peer support risks assuming equal levels of emotional competence among participants, which may not hold in practice. Movement toward a cultivated gaze was observed when structured tools and dialogic practices were introduced, signaling a shift from identity‐based legitimacy toward dispositions developed through systematic engagement. However, sustaining this movement requires organisational commitment to allocate time for reflection.

Intervention 2, aimed at fostering collaborative learning and onboarding, was dominated by a cultivated gaze, emphasising structured interaction and reflective dialogue rather than innate qualities. While this gaze supports interpretive judgement and professional growth, traces of a trained gaze appeared through procedural frameworks. Transitioning from a trained to a cultivated gaze requires strengthening IR without increasing SubR, shifting legitimacy from procedural mastery toward interpretive judgement.

Intervention 3, designed to train group leaders as internal supervisors, exemplified a trained gaze, privileging codified procedures and technical skills while minimising identity‐based legitimacy. Case‐based reflection introduced opportunities for movement toward a cultivated gaze, but this shift depends on creating sustained spaces for dialogue beyond procedural compliance.

Intervention 4, focused on communication skills in dementia care, reflected a trained gaze, emphasising task‐based learning and observable behavioural change. Legitimacy was conferred through procedural mastery rather than dialogic engagement, though supervisors occasionally encouraged interpretive sensitivity to patient context. Moving toward a cultivated gaze would require deliberate integration of reflective debriefing and interpretive discussion; otherwise, technical training risks constraining epistemic development.

### 4.2 The Gazes and Their Implications

Cultivated gaze enables interpretive judgement through structured engagement, in contrast to the trained gaze's emphasis on procedural mastery. Social gaze legitimates knowers based on identity and shared experience, which may limit epistemic development unless complemented by cultivated dispositions formed through guided immersion in practice. Movements from social → cultivated and from trained → cultivated gaze observed in this study suggest that supervision can reconfigure knower legitimation by comparatively strengthening IR while maintaining or reducing SubR. These transitions, however, are not automatic; they require dialogic spaces, institutional support, and pedagogical strategies that balance technical competence with interpretive judgement.

Without such conditions, interventions risk becoming confined to either identity‐based validation (social gaze) or procedural compliance (trained gaze), thereby constraining deeper reflective practice. Sustaining cultivated dispositions demands a cultural shift that prioritizes reflective inquiry alongside operational efficiency, which is a challenge in resource‐constrained clinical environments.

The municipality's approach aligns with broader reflective practice principles while operationalising them through the cultivated gaze, which privileges interpretive engagement over procedural adherence. At the same time, the emphasis on a social gaze demonstrates how shared experiences are used to foster a sense of community among staff. This finding supports literature highlighting the value of interpersonal reflection within professional relationships [39] and echoes studies showing that mentorship and collaborative dialogue enhance professional understanding [18, 44]. The relational dynamics associated with a social gaze help mitigate the isolation often experienced by healthcare workers, thereby reinforcing collaborative learning environments [4, 5, 6, 7].

Our analysis revealed a nuanced interplay among the gazes, especially between the trained gaze and the cultivated gaze. While the trained gaze provided structured frameworks and tools beneficial for specific competencies, reliance on these methods without integrating emotional engagement or interpersonal learning risks oversimplifying the complexities of care, particularly in emotionally demanding situations. This finding is consistent with existing literature that acknowledges barriers to effective reflective practice, such as time constraints and reliance on procedural norms [29, 30].

Furthermore, the presence of a weaker social gaze in Interventions 3 and 4 revealed that existing hierarchies could inhibit engagement, limiting participation from less dominant voices. While social gaze can foster solidarity by emphasising shared identity, it legitimates knowers based on subjective attributes rather than structured engagement. This may fragment epistemic progress if not complemented by cultivated dispositions [39]. Future training programs might benefit from intentionally fostering conditions where the social gaze is more pronounced, thus addressing fragmentation in understanding the knowledge constructed within the supervision framework.

Concrete supervision models vary in numerous aspects, such as methodology, participant roles, time frames, and frequency. A critical element of supervision is that it often occurs within a work context, where the primary focus is on delivering the expected outputs that align with organisational goals. This workplace pressure can lead supervision and learning, which are activities essential for fostering reflective practices, to be deprioritized amidst the busyness of daily operations, even though this is not the intention of the staff.

### 4.3 Reflections on Movements Across the Social Plane

Mapping the four supervision models on Maton's social plane shows shifts from identity‐based legitimation (social gaze) and procedural mastery (trained gaze) toward dispositions cultivated through structured interaction (cultivated gaze). These movements suggest the municipality aimed to democratise knower legitimacy by reducing reliance on innate or social attributes and emphasising learnable, socially mediated dispositions. Sustaining cultivated gazes, however, requires institutional commitment to dialogic spaces and time allocation; without these, interventions risk reverting to procedural compliance or identity‐based validation, limiting reflective depth.

#### 4.3.1 Limitations

Both participants and external consultants were selected by the municipality, which may have influenced the perspectives represented in the data. The consultants' professional backgrounds and approaches likely shaped the supervision practices, and the participants' willingness to engage may reflect pre‐existing attitudes toward supervision rather than the full diversity of staff experiences.

The municipality played a central role in designing the project and determining which external consultants facilitated the interventions. This strong institutional influence may have affected the emergence of supervision models and the legitimation practices observed, limiting the generalizability of findings to other organisational contexts.

While LCT provided a useful lens for analysing knower legitimation, its application to clinical supervision is relatively novel. The theory's abstraction can make operationalization challenging, and alternative frameworks might capture other dimensions of supervision, such as power dynamics or emotional labor, more explicitly. We did not identify examples of the born gaze, aligning with the intervention's emphasis on learnable competencies.

#### 4.3.2 Future Research

The barriers identified in this study such as limited access to supervision and time constraints mirror challenges reported in previous research [29]. Future interventions should therefore explore flexible supervision models that embed reflection into routine practice rather than treating it as an additional task. Approaches that foster collaborative and critical reflection may strengthen collective learning environments, as highlighted by Rudolph et al. [44].

Applying LCT in future studies offers promising avenues for understanding how different forms of gaze shape learning environments and professional identity. Our findings suggest that visualising movements across the social plane of gazes could provide valuable insights into how practitioners transition between orientations (e.g., from trained to cultivated gaze). Developing diagrammatic representations of these pathways would allow researchers and educators to assess the effectiveness of supervision practices and identify conditions that enable such shifts.

Finally, future work should critically examine the role of institutional influence such as municipalities selecting external consultants in shaping supervision models. Investigating how organisational priorities interact with pedagogical frameworks will be essential for designing supervision practices that are both context‐sensitive and equitable.

## 5 Conclusion

Our mappings illustrate how different supervision models shape what is considered a “legitimate knower” in primary care. Although all interventions aimed to enhance reflexivity, the dominant gaze in each model reveals distinct pedagogic and epistemic priorities within professional development. This analysis advances understanding of how supervision can be tailored to foster authentic reflective practice by balancing interpretive dispositions (cultivated gaze), identity‐based support (social gaze), and procedural mastery (trained gaze).

The gaze embedded in a given supervision model significantly shape the conditions under which reflexivity is recognized and sustained. Some forms of supervision create conditions that encourage practitioners to examine their thoughts, emotions, actions, and assumptions, thereby deepening reflexive capacity. Others may inadvertently constrain this process, limiting practitioners' ability to navigate supervisory relationships and recognise underlying power dynamics. Consequently, the supervisory approach can either enable or inhibit meaningful reflection, with direct implications for practitioners' ongoing professional development.

## Author Contributions

**Anette Lykke Hindhede:** conceptualization, methodology, investigation, data curation, writing – original draft, writing – review and editing. **Helle Hindhede Hald:** conceptualization, writing – review and editing. **Christina Andersen:** visualization, writing – review and editing. All authors reviewed the manuscript.

## Funding

The formative evaluation research was funded by the City of Copenhagen.

## Conflicts of Interest

The authors declare no conflicts of interest.

## Data Availability

The data that support the findings of this study are available on request from the corresponding author. The data are not publicly available due to privacy or ethical restrictions.
